# Natural Gum-Based Functional Bioactive Films and Coatings: A Review

**DOI:** 10.3390/ijms24010485

**Published:** 2022-12-28

**Authors:** Arushri Nehra, Deblina Biswas, Valentina Siracusa, Swarup Roy

**Affiliations:** 1 School of Bioengineering and Food Technology, Shoolini University, Solan 173229, India; 2 Department of Chemical Sciences, University of Catania, Viale Andrea Doria 6, 95125 Catania, Italy

**Keywords:** natural gums, polysaccharides, edible polymers, coating and film, active packaging

## Abstract

Edible films and coatings are a current and future food packaging trend. In the food and envi-ronmental sectors, there is a growing need to understand the role of edible packaging and sus-tainability. Gums are polysaccharides of natural origin that are frequently utilized as thickeners, clarifying agents, gelling agents, emulsifiers, and stabilizers in the food sector. Gums come in a variety of forms, including seed gums, mucilage gums, exudate gums, and so on. As a biodegradable and sustainable alternative to petrochemical-based film and coatings, gums could be a promising option. Natural plant gum-based edible packaging helps to ensure extension of shelf-life of fresh and processed foods while also reducing microbiological alteration and/or oxidation processes. In this review, the possible applications of gum-based polymers and their functional properties in development of edible films and coatings, were comprehensively dis-cussed. In the future, technology for developing natural gum-based edible films and coatings might be applied commercially to improve shelf life and preserve the quality of foods.

## 1. Introduction

Balanced nutrition is essential to maintaining optimal health in an individual, and can be obtained through food. Hence, food plays an important role in everyone’s lives. In this current era of globalization, food packaging has emerged as a most exciting and pro-ductive aspect of the food industry [1,2]. Food waste is currently a key concern, as it con-tributes to a high amount of food loss and has a negative influence on national resources and economic progress. Furthermore, food oxidation plays a significant role in degrading food quality due to its deteriorating effects. It reduces the nutritional quality and flavor of food, raises toxicity, and alters textural qualities. Thus, a primary focus of the food indus-try is to preserve food quality and wholesomeness, thus gaining in consumer acceptability. One viable alternative to dealing with food waste and plastic packaging problems would be through incorporating edible film- and coating-based food packaging [3,4,5,6].

The global production of packaging materials rises by roughly 8% every year. Over 90% of used plastics are accumulating in landfills, while <5% are recycled, resulting in a huge environmental threat [7,8]. Inventors have focused on the development of appropri-ate tools to resolve these concerns. Attempts have involved various tactics, such as in-creased safety, preservation quality, and recycling [9,10]. Biodegradable packaging is an attractive alternative to traditional plastics due to its sustainability, renewability, and nontoxicity. Thus, a variety of biopolymers have been used to produce materials for envi-ronmentally friendly food packaging [11,12,13,14,15]. Recently, demands have increased for food packaging that does not contribute to pollution, that is produced using environmentally friendly methods, and that remains affordable. Coatings and edible films are key packag-ing types for edible materials [4,16,17,18,19].

Food packaging extends shelf life by reducing unwanted variations in food and safe-guarding food from microbiological infection, moisture loss, and exterior damage. In this context, active food packaging (AP) technology is a promising approach [20,21,22,23]. Edible films and coatings can be used as postharvest treatments to protect the quality of foods while lowering the amount of nonbiodegradable packaging materials used. As a result, edible films and coatings fabricated from hydrocolloids (e.g., chitosan, carrageenan, pul-lulan, hydroxyl propyl methyl cellulose, alginate, etc.) are commonly used to maintain the quality of foods [6,24,25,26,27,28]. To make edible films, natural gums could be a promising alter-native, as they are biocompatible, inexpensive, nontoxic, and readily available [29,30]. Natural gums have attracted considerable interest recently due to their unusual rheologi-cal qualities and structural variety. Gum hydrocolloids, often called gum polysaccharides, are common and adaptable polymers utilized to collect materials with varying structural and functional characteristics [29]. Several novel gums have been reported in the litera-ture in recent years from various sources [31,32].

Gum-based edible packaging is already known to preserve the quality of fruits and vegetables after harvest [33]. Plants, microbes, and animal tissues are all sources of gums. However, plant-derived gums are the most popular [34]. Plant-derived gums are readily available. Furthermore, two or more gums can be mixed together to offer synergistic bene-fits [29]. In gums, plant seed gums like guar, locust bean, tara, and tamarind were used to extract or isolate polysaccharides [35]. Polysaccharides-based gums are generated from the endosperm of many plant seeds (mostly leguminosae). The majority of polysaccharides gums are galactomanans. These are polysaccharides that are primarily made up of the monosaccharides mannose and galactose. Depending on the plant origin, mannose com-ponents from linear chains are coupled with galactopyranosyl residues as sidechains [36].

Gums can bind water and produce gels. Mucilage gums, seed gums, exudate gums, and other types of gums exist [32,37]. Gums come from a variety of plant parts, e.g., seed epidermis, leaves, and bark [37]. Some plant gum exudates, such as karaya, Ghatti, and tragacanth, have been well documented in recent decades. Gum Arabic has been used for over 5000 years [4,38]. Natural gums have generated interest in fruit and vegetable coat-ing applications [39].

There have been few literature reviews on gum, gum extraction methods, or methods of dealing with gum structural modification. There have been studies on gum-based film and coatings and their application in food systems, however [35]. Previously, Khezerlou et al. reviewed fabrication of edible films and coatings using plant gums [35]. The use of gum coating on food products has been reviewed [29]. Recently, gums have been used in active and intelligent food packaging, i.e., film and coating [32,37,40,41]. Although some review articles have been published on natural plant gum-based films and coatings, there were, as of this review, no up-to-date reports available regarding the use of gums in food packaging. Therefore, in this review, we introduced various types of gums, their sources and properties, and finally, their utilization in food packaging film and coatings. Consequent-ly, the application of various gum-based bioactive functional composite film and coating in fruits, vegetables, and animal food product packaging was discussed comprehensively.

## 2. Types of Plant Gums

There are various types of plant-based gums. Gums and mucilage are mainly poly-saccharides. Gums are known as polyuronides and contain various salts of potassium, magnesium, calcium, and so on [35]. Mucilages are mainly sulfuric acid esters of polysac-charides. Galactose and arabinose are commonly found sugars in gums and mucilage [29]. The details of various types of gums will be briefly discussed below.

### 2.1. Seed Gum and Mucilage

Gums are derived from a variety of plant components. Some gums come from the seed epidermis, whereas others are derived from plant leaves. Gums are pathogenic con-stituents that are produced by plant impairment or unfavorable situations. Mucilage is a naturally metabolic product generated in the cell. Mucilage is insoluble in water. Almost all plants and certain microbes create mucilage, a thick, sticky material. Both gums and mucilage are plant hydrocolloids; hence, they have certain similarities. They are likewise made up of a combination of transparent amorphous polymers and monosaccharide pol-ymers, as well as uronic acid [37]. Mucilage is widely used in the food industry, owing to its exceptional functional properties (e.g., antimicrobial, antioxidant, water-holding, oil holding, etc.) which are beneficial for food packaging film and coatings [35,37]. However, the films formed from mucilage films are fragile and have poor mechanical properties [35].

#### 2.1.1. Guar Gum

Guar gum is a galactomannan originating from the seed of the plant Cyamopsis tetragonolobus [42,43]. Guar gum is made by separating the endosperm from the hull and germ [44]. It is a high molecular weight, odorless polysaccharide attained from the guar plant that has a white to yellowish-white color. The guar plant grows to a height of 0.6 m, with pods ranging in length from 5 to 12.5 cm [31]. This plant is a largely sun-loving plant that can withstand high temperatures, but it is vulnerable to cold. Guar gum powder is the most common form used as a food ingredient. Guar gum is similar to locust bean gum in that it is made up mostly of the complex carbohydrate polymer galactose and mannose. However, the amounts of these two sugars differ in these gums. India produces 80% of the world’s guar, with Rajasthan accounting for 70% of the crop. Guar is cultivated in north-ern provinces of India, including Rajasthan, Gujrat, Haryana, and Punjab, and India is the global leader in guar production [45]. Guar gum-based films are known for their great mechanical strength, good barrier qualities, and antibacterial or germ resistance [31]. Because of its long polymeric chain and high molecular weight relative to other forms of gums, guar gum is an excellent choice for making edible coatings. It is a galactomannan with a mannose backbone ((14)-linked -D-mannopyranose) and galactose side groups ((16)-linked -D-galactopyranose).

#### 2.1.2. Locust Bean Gum

Locust bean gum (LBG) is made from the seed endosperm of the carob tree’s fruit pods. These are botanically known as *Ceratonia siliqua* L. and are found in Mediterranean countries. As a result, locust bean gum is often referred to as carob gum. The husk, endosperm, and germ are the three sections of the carob seed. LBG is used in food, paper, textiles, oil well drilling, and in the cosmetics sector [45]. It is a neutral polysaccharide comprised of mannose and galactose. The seeds are primarily made of galactomannan, which accounts for around 80% of the total weight. The remaining 20% is proteins and impurities. The protein concentration of LBG is roughly 32% albumin and globulin, and the rest is glutelin and impurities (ash and acid-insoluble materials) [46]. Among natural polysaccharides, LBG is a promising alternative for food packaging. LBG is only weakly soluble in cold water. In order to attain full hydration and maximal viscosity, an LBG solution must be heated up to a certain temperature. It can produce films and coatings with high mechanical and water vapor barrier characteristics. LBG has been explored for its potential applications in film production and coating applications [47]. It can also be used to stabilize dispersion and emulsion in the food sector as a fat replacement in several dairy products [34].

#### 2.1.3. Tara Gum

Tara gum, commonly known as Peruvian carob, is obtained from the seed endosperm of the *Caesalpinia spinosa* tree [48]. The primary constituent of this gum is galactomannan polysaccharides [49]. Tara gum is a commonly used food additive [50]. It is also used as a thickener and binding agent. The water-binding ability of Tara gum makes it perfect for rapid hydration-forming thick colloidal solutions. It can be used as a thickening agent or viscosity modifier. Additionally, it is used as a polymer matrix for food packaging applications.

#### 2.1.4. Basil Gum

Basil (*Ocimum basilicum* L.) is widely grown in India’s Himalayan states of Jammu and Kashmir. In aqueous conditions, the seeds of this plant create mucilage. Mucilage is produced and is closely linked to the seed core, providing a large amount of polysaccharides and soluble fiber [51]. Basil is an aromatic plant that is commonly used to give food a distinct aroma and taste. To use as a spice, the leaves can be used fresh or dried. Food additives can be made from essential oils taken from fresh plants and flowers. Basil seed gum (BSG) has great potential in the food sector as a gelling, foaming, thickening, binding, fat replacement, and reducing agent [52].

#### 2.1.5. Fenugreek Gum

Natural fenugreek gum is produced by the *Trigonellafoenum graecum* plant (Family Leguminosae). Northern Africa, Canada, Western Asia, and India are among the countries that grow it. It can be used as a spice, a vegetable, or a medicinal herb, among other things [53]. The leaves, seeds (whole and gum), chemical components (such as hydroxy isoleucine), and immature shoots are known to possess antioxidant activities [54]. It is also applied as a food binder, glue, and emulsifier in food products [53]. More significantly, it has been used to create healthy, nutritious extruded and baked products. Because of its dietary fiber, protein, and gum content, it is utilized as a food stabilizer, glue, and emulsifying agent. Fenugreek is made up of 23–26% fenugreek, protein, 6–7% fat, and 58% carbohydrates (approximately 25% of which is nutritional fiber) [55]. Dietary fiber, particularly soluble fiber, may be found in a variety of foods and beverages, including cereal bars, yogurts, and nutritious beverages. Soluble fiber powder or total dietary fiber powder can be mixed with fruit juices, spices, and other spice blends [56].

### 2.2. Exudate Gum

Plant exudate-based gums originate from the bark and branches of trees to protect them from environmental and microbiological harm. The earliest forms include exudates gums, which are regularly utilized as thickeners, stabilizers, rheology modifiers, soluble fiber, and fat replacers [44].

#### 2.2.1. Gum Ghatti

The extruded gum from the *Anogeissus latifolia* tree, commonly known as India Gum, is known as gum ghatti (GG). Gotifilia is the term given to a newly produced GG, a spray-dried powder made from specially selected high-grade GG. It has a uniform color and is extremely soluble. The high emulsification characteristics of GG is a key attribute. GG’s capacity to emulsify is superior to that of gum Arabic or any other natural gum. It may form stable emulsions at concentrations as low as 25% of those of gum Arabic [57]. Due to its component glycoprotein, GG could potentially be useful in manufacture of films. It is commonly used in the food industry as a thickener, stabilizer, and emulsifier [58]. It has been used in India since ancient times due to medicinal and related characteristics that make it beneficial for use in food items. It is found in numerous writings such as Indian (Ayurveda) and Greek (Unani) medical systems. It is used as a wellness product in some parts of India, or as a mark of status and richness [32].

#### 2.2.2. Persian Gum

Persian gum (PG) is derived from the *Amygdalus scoparia*. Since PG is less expensive than other natural hydrocolloids, it is a possible alternative to other gums. It is comprised of approximately 90% polysaccharides, mostly galactopyranosyl and arabinofuranosyl, based on the dry weight. PG can normally be dissolved in water at 25–30% which makes it a good chemical for the production of film-forming solutions [59]. Currently, it has the potential to be used as a suspending or emulsifying agent in foods, medicines, and other sectors [44].

#### 2.2.3. Tragacanth Gum

Tragacanth gum (TG) is produced by wounds in some plants. *Astragalus gummifer* and other *Asiatic Astragalus* species generate the gum as a dry exudate from their stems and branches [40]. It is commonly utilized as a natural thickening agent and emulsifier. Additionally, it possesses outstanding thermal stability, great solubility, and good rheological behavior [60]. It is an excellent emulsifier which enhances aqueous phase viscosity [61]. TG is known to be used as a thickening agent in a variety of foods, including sauce, ice cream, jelly, salad dressing, syrup, confectionery, and mayonnaise [40]. In the presence of water, TG expands and forms a polymeric molecular network. This eventually stabilizes the aqueous and serum phases of food items and increases their viscosity. TG is also utilized as a binder in a variety of confectionary products (i.e., candies). Subsequently, it is used in ice cream to achieve a smooth texture as well as to hinder ice crystal development during storage [32]. In Table 1, types of gums used in fabrication of bio-based edible film are presented.

## 3. Preparation of Natural Gum-Based Films and Coatings

**Figure 1:**
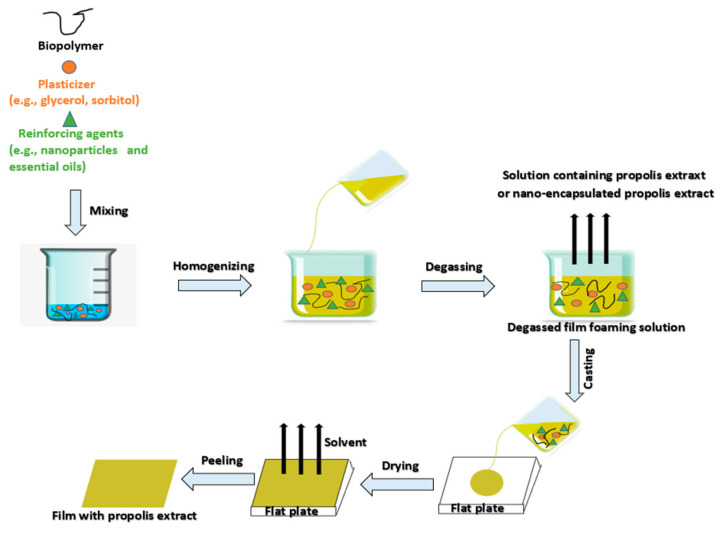
Schematic representation of various stages in a film casting process (Modified from Yong & Liu et al., 2021 [66]).

**Figure 2:**
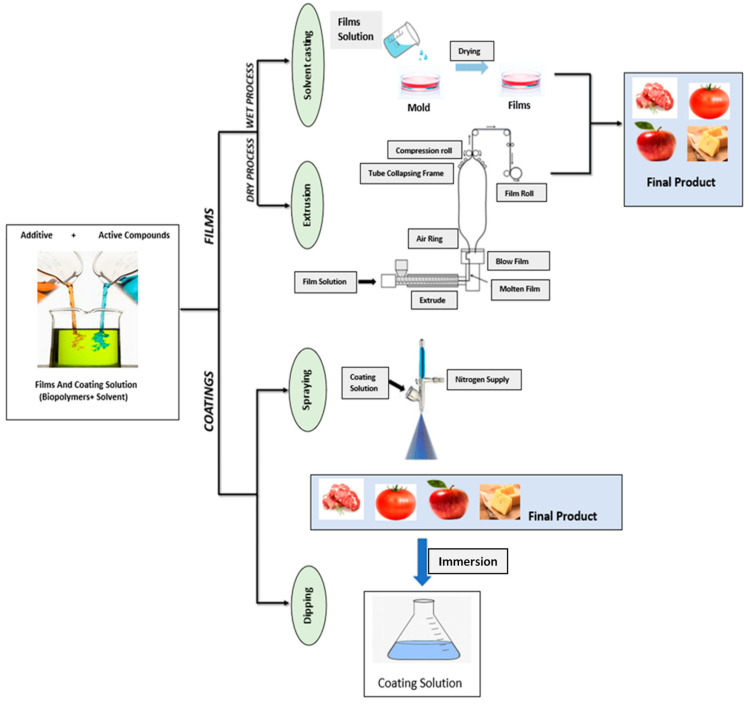
Preparation process of films or coatings for active food packaging application (Modified from Ribeiro et al. (2021) [67]).

The fabrication of natural gum-based films is primarily performed by relatively simple solution casting techniques. Various stages of film casting processes are schematically represented in Figure 1, while an overview of preparation process of films or coatings for active food packaging application is illustrated in Figure 2. Furthermore, the preparation of various natural gum-based films is briefly described herein, and some of the results are tabulated in Table 2.

### 3.1. Guar Gum

A total of 150 mL of film-forming solutions were put onto Teflon plates (15 cm × 15 cm) lying on a flat surface to make the films. The dried films were peeled away from the surface of the casting. In a controlled temperature and humidity chamber, films were equilibrated at 23 °C and relative humidity (RH) of 50% to measure barrier and mechanical characteristics [62].

### 3.2. Locust Bean Gum

For complete solubilization of LBG in an aqueous solution, it was heated for 1 h at 80 °C using a magnetic stirrer. The gum solutions were left to stand overnight at 4 °C after dissolution. Before casting into plates, film-forming solutions were centrifuged to eliminate air bubbles. Each gum solution was poured into Teflon plates and dried in an air oven. They were then carefully peeled off the plates and stored with saturated Mg (NO_3_)_2_ at 23 °C until they reached a constant weight at RH of 52.80 ± 0.20% [47].

### 3.3. Tara Gum

TG (0.75%) solution was made in distilled water using agitation at 45 °C. The plasticizer (a 1:1 combination of sorbitol and glycerol) was then added. After that, ultrasonic treatment was used to eliminate any remaining air bubbles in the solution and it was cast in petri plates [63].

### 3.4. Basil Seed Gum

A total of 70 g of mucilage was mixed with 30 g kg^−1^ (based on BSM weight) glycerol as a plasticizer to make basil seed mucilage (BSM) film. Then, depending on the original weight of BSM, 30 g kg^−1^ of TA, MA, and SA were added. At 60 °C, the solution was agitated continuously for 45 min. BSM was made by putting the mixture onto a polypropylene plate and drying it for 10 h at 40 °C in a hot air oven. To complete crosslinking, the BSM film was baked in the oven at 150 °C for 10 min. Before characterization, the dry BSM film was peeled off and stored [64].

### 3.5. Fenugreek Gum

FSG was dissolved in distilled water containing glycerol for 3 h at 1000 rpm at 65 °C. Nano clay (2.5, 5, and 7.5%) was then gently mixed into the FSG solution. The nanocomposite films were made by pouring the film forming solutions onto Teflon plates and drying them for 24 h at 45 °C [55].

### 3.6. Ghatti Gum

GG films were made by mixing 0.75 and 1.0% GG in water for 15 min at 25 ± 2 °C with steady stirring. In a thermostatic bath water, they were heated at 40 °C for 1 h. After cooling to room temperature, plasticizer was mixed, and then cast on Petri dishes [58]. Finally, dried films were immersed in crosslinking solutions (1.5% CaCl_2_ + 1% citric acid) for 5 min to yield crosslinked films.

### 3.7. Tragacanth and Persian Gum

Tragacanth and Persian gum granules were mixed in water and heated to 60 °C for 30 min until the particles were completely dissolved. Following that, glycerol was added and agitated for an additional 15 min and then cast to make film [65].

### 3.8. Mucilage

Various types of mucilage were already used for the fabrication of film [77,78,79,80]. Recently biodegradable film was developed based on *Pereskia aculeata* Miller mucilage [81]. The film-forming solution was made by dissolving 1.5, 1.8, and 2.0% of the mucilage in water and kept for 12 h for hydration. Thereafter homogenization was used at 12,000 rpm for half an hour and then varying content of glycerol (20–25% (*w*/*w*)) was mixed and again stirred for 10 min. The film forming solution was casted in Teflon coated surface to fabricate the film.

## 4. Various Film Forming Properties of Natural Gums

Primarily, there are two key types of packaging: edible films & coatings. The films are mainly utilized for edible packaging, wrapping over the food surface whereas the coating is directly used on the food surfaces [11]. Casting and extrusion are the most commonly used tools to fabricate film. On the other hand, there are many methods, such as dipping, panning, spraying, etc., for coatings [13].

The film casting process uses a wet chemical method for developing film. It is the most commonly used method for making film at lab scale. In this method, the biopolymers are solubilized in a solvent, and in this regard, water and ethyl alcohol are most commonly used. The completely soluble biopolymer solution is poured into the mold and dried until all the solvent is removed to make a film [22]. The primary advantage of this method is easy handling without any equipment and cost effectiveness. However, it has many limitations, including a long processing time, limited forms, etc. On the other hand, the extrusion technique is a commercially-applicable dry method for making film. In this method, generally, no solvent is used. It includes melting of biopolymers and mixing with other ingredients to produce film of the prescribed thickness and shape. The extrusion process in temperature sensitive and as a result, biopolymers which are highly sensitive to temperature cannot be used in the extruder [13]. Extrusion is an efficient and high-performance method for commercial purposes but in this process, only temperature-tolerant polymers can be used. Additionally, the cost of equipment is high, another major drawback of this method.

In coating, dipping is the most commonly-used method in lab scale. It includes immersion of the food system in the coating formulation and generation of a thin layer on the food product [28]. A thick coating is sometimes disadvantageous for respiration of food. Another widely-applied technique is spraying, in which liquid solution is sprayed in the form of small droplets on the surface of the food items. There are different types of spraying methods, such as air spray atomization, pressure atomization, etc. [28]. This method produces uniformly thick coating on the food surface but the highly viscous coating formulation is difficult to spray. Panning is another suitable and efficient method for coating of foods, where in a large pan of food is coated while spinning. Using this method, large quantities of food items can be coated easily [28].

While edible films and coatings lack in several essential packaging characteristics (e.g., mechanical strength and water barrier capabilities, lack of functionalities), they provide certain unique features (e.g., biodegradability, sustainability) to food packaging. There are solutions to extant problems. Mixing of bioactive components like essential oils (EOs) in the gum-based edible film can be useful to improve physical and functional properties (e.g., antibacterial and antioxidant activities). The release of oil-soluble compounds from the edible film into the mobile lipid phase of fatty meals might provide extra nutritional advantages while also preventing oxidative rancidity and microbiological deterioration. A decrease in film hydrophilicity was predicted with the addition of essential oil, crosslinking, or bioactive compounds. The addition of orange oil, along with curcumin, induced antibacterial properties in GG films [68].

In a study, Martins et al. developed LBG- and kappa-carrageenan (k-car)-based edible films with specific qualities [70]. The mixing of k-car to LBG increased the films’ barrier characteristics, resulting in a reduction in WVP. Furthermore, as compared to carrageenan and LBG films, the carrageenan/LBG blend films had a higher tensile strength (TS). The same authors studied the effects of organically modified clay Cloisite 30B (C30B) on the same composition of film [69]. The authors reported that, with the increase in clay content, strength and flexibility were improved remarkably. Moreover, the authors reported that, as quantity of C30B in the film formulation was increased, the antibacterial activity toward *L. monocytogenes* improved. Carrageenan/LBG–C30B showed antibacterial action.

Gahruie et al., observed that, in the presence of *Z. multiflora* essential oils nanoemulsion, the mechanical characteristics of the film improved and they showed increased antibacterial activity by reducing the particle size of the nanoemulsion [72]. *Z. multiflora* essential oils (ZMEO) were effectively added to basil seed gum films in order to fabricate next-generation active packaging materials with enhanced antimicrobial properties. In another study, when oregano essential oil was added to basil seed gum edible film, it resulted in a film with potential antibacterial and antioxidant properties [71]. Memis et al. used a nano clay to fabricate FSG films [55]. The nano clay improved the barrier properties of the films. FSG-based nanocomposite films have good mechanical characteristics and antibacterial activities, which could be promising for use in food packaging.

A combination of sodium alginate and GG was investigated in order to enhance the performance of biodegradable SA film [73]. The film had improved mechanical and barrier properties. In addition, the mix film’s light barrier qualities were increased by 65.17%. Khezerlou et al. reported a film made from sodium caseinate (SC), *Zingiber officinale* extract (ZOE), and PG [74]. The addition of ZOE resulted in a considerable improvement in tensile strength. However, the presence of ZOE and the PG reduced elongation at break (EB). Furthermore, the addition of ZOE improved hydrodynamic properties, but the presence of PG increased opacity. Tonyali et al., studied the effects of whey protein isolate (WPI) on tragacanth gum-based film [75]. The results showed that combining WPI and TG in film formulation resulted in elastic, less soluble films with reduced water permeability and less transparency. The addition of Plantago seed mucilage in carrageenan gum led to reduction in mechanical strength and increase in flexibility of the film. The antioxidant activity and crystallinity of the carrageenan-based film was improved [76]. The blending of chia seed mucilage (2.5%) on levan-based film was studied [82]. The author reported that the presence of mucilage had a good impact on the mechanical and barrier properties of the levan-based film. The transparency of the film was reduced, but the antioxidant activity and antimicrobial performance of the film were significantly enhanced.

The biodegradable film fabricated using *Pereskia aculeata* Miller mucilage was opaque and the films mechanical properties was low (TS = ~1–5 Mpa) [81]. The moisture barrier properties of the films based on mucilage were higher compared to synthetic film and the water solubility is low. The film also showed good thermal stability. The authors inferred that both mucilage and glycerol content affected the physical properties such as mechanical, barrier, and thermal properties of the film. In another work, quince seed mucilage (1%) based functional packaging film was developed adding thyme essential oil [83]. The film exhibited good mechanical and barrier characteristics excellent antioxidant activity and inhibited the growth of *Shewanella putrefaciens, Listeria monocytogenes* and *Staphylococcus aureus*.

Dick et al., 2015 studied the fabrication of Chia seed mucilage edible film [84]. 1% mucilage and varying content of glycerol (25, 50, 75% (*w*/*w*)) was used to produce the film. The fabricated films presented high solubility, transparency, and strong UV-light barrier properties. With increasing glycerol content, the mechanical strength of the film was reduced while the elongation at break and water vaper permeability increased. The blend film of chia seed mucilage in whey protein isolate (WPI) was studied by Muñoz et al., 2012 [85]. The authors used chia mucilage in blend (1:3 or 1:4) with WPI for the development of film. They reported the formed film showed good mechanical properties and high water vapor barrier properties. In another work edible and biodegradable film was developed using mucilage from *Opuntia ficus-indica* [86]. The mucilage-based films’ physical properties were reported to improve by adding pectin. Araújo et al., studied the Okra mucilage based edible packaging film and reported the characteristics of the film was analogous to the other polysaccharide-based film [87]. The physical properties of the film such as water vapor permeability, solubility, thermal and mechanical properties, etc. were improved by mixing with starch. Very recently the cactus (*Cylindropuntia fulgida*) mucilage has also been used to fabricate biopolymer based antimicrobial packaging film [88]. The authors used Cactus mucilage and gelatine as biopolymers while *Euphorbia caducifolia* extract as an antimicrobial agent. The various physical properties such as water solubility, the water vapor barrier properties, flexibility as well as antimicrobial activity of the films were meaningfully improved in presence of 20% extract.

## 5. Application in Food Preservation

Various gum-based edible coating and films are effective to delay aging and extend the storage life of various foods. Natural hydrocolloid-based edible coatings and films provide extra protection to foods.

Dipping, coacervation, and spraying are a few of the available methods for applying edible coatings on food [89]. Each method has a number of reported benefits and drawbacks, and the success of each method is greatly determined by the traits and qualities of the items to be coated, as well as the coating’s physical characteristics (viscosity, surface tension, density) [90]. For instance, in a study, while using the dipping approach, the outer layer of the meal was seen to be diluted by coating suspensions. Consequently, functional properties of the coating are reduced by fruits and vegetables. Edible finishes are often applied in single layer coatings on food using the dipping technique [91]. Natural gum edible coatings provide a promising way to expand the quality of foods while also extending their shelf life [29]. The use of natural gum-based films and coating on food preservation is briefly presented in Table 3.

**Figure 3:**
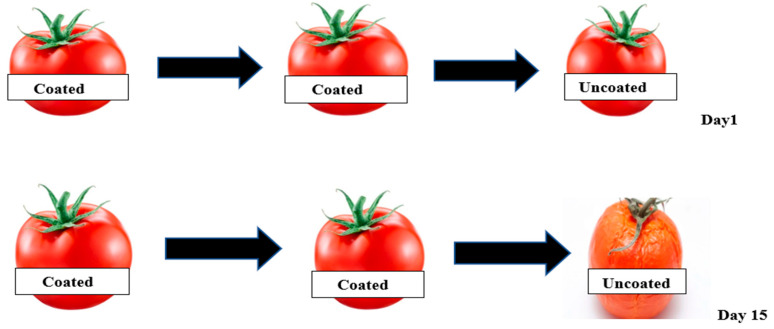
Effect of storage time (day 1 and day 15) on Roma tomatoes coated with guar gum (C) and uncoated (UC) at room temperature (22± 2 °C) (Modified from Ruelas-Chacon et al., 2017 [92]).

GG was used to delay the ripening of Roma tomatoes by reducing their respiration rate [92]. The results indicated that GG coating not only preserved firmness but also enhanced postharvest quality when stored at room temperature. The GG coating was transparent and stuck effectively to the surface of the Roma tomato. During the 20-day storage period, all tomato fruits shrunk, but the coated ones shrunk more slowly than the uncoated ones. The GG coating was biodegradable, easy to apply, and less costly than other hydrocolloids and commercial waxes. Therefore, it could be applied large-scale to extend the shelf-life of Roma tomatoes (Figure 3).

Mangoes are seasonal fruits with a short post-harvest life. Their storage life is mostly determined by the mango fruit variety chosen and the storage conditions. When kept at 13 °C, the shelf life might be as long as a week. Mango fruit losses of 20–30% are recorded annually, amounting to 3000 tons (~28 million USD), owing to incorrect handling, insufficient storage and poor strategies after harvest [93]. Various techniques have been used to increase the life span of mangoes in normal and cold storage environments. Mangoes coated with GG infused with EOs had a 24-day shelf life. The hardness of the mangoes diminished as the storage period progressed. However, the delicious scent of the raw mangoes remained. The fruits’ skin color altered from greenish to yellowish, indicating that they were fully ripe.

The use of LBG coating on sausages led to a prominent reduction in moisture loss [94]. Coating lowered the rate of respiration, reduced the oil content, and prolonged the life span of the meat. The coated samples could be stored for up to two weeks during cold storage at 5 °C. TG reduced mass loss, maintained firmness, reduced color change, and inhibited mold and yeast development in peaches [95]. The best results were obtained using Tara gum in combination with citric and ascorbic acids, as well as sodium chloride. Tara gum reduced mass loss, maintained firmness, reduced color change, and inhibited mold and yeast development. As a result, this gum showed great potential an edible coating substance.

The use of BSG coating as a polysaccharide-based coating on fresh strawberries improved their physicochemical, sensory, and microbiological qualities during cold storage [96]. Furthermore, adding echinacea extract to the BSG coating composition improved the quality of fresh strawberries in a synergetic manner. Microbial counts (yeast and molds) decreased as the content of Echinacea extract increased.

Recently it was reported that the optimal coating composition was able to increase the postharvest quality of guava fruit [97]. The response surface approach was shown to be a useful statistical tool for separating the interacting effects of independent variables. Weight loss and TSS were considerably decreased when edible coatings based on FG and Guar galactomannan were used. Furthermore, during storage, the covered fruit was fresher, firmer, and lower in TA. Weight loss was found to be 1.71% and 2.11%, firmness to be 0.72% and 2.14%, TSS to be 1.02% and 1.44%, pH to be 0.83 and 1.36, and acidity to be 1.03% and 1.44% in edible coatings, respectively. Coated guava showed a significant reduction in weight loss and maximal firmness retention. TSS enhanced in all treatments up to a specific storage duration and then declined as the storage period extended. However, pH increased while acidity decreased significantly. The edible coating of guava may be enhanced significantly by integrating Guar galactomannan and FG.

Grapes were coated with chitosan-based formulas with and without GG to increase postharvest quality [57]. Weight loss, acidity, pH, etc., of grapes were all improved when gum-ghatti was added to the chitosan solution. During two months of cold storage, coatings slowed down variations in ascorbic acid and enhanced polyphenol oxidase antioxidant enzyme activity. The chitosan-gum-ghatti-based coatings retained the bioactive components in grapes. The appropriate sort of chitosan-GG coating on grapes could help to improve its life span during transportation.

In comparison to untreated controls, the use of Kurdi Gum (KG) and PG solutions considerably enhanced the sensory features of bananas, and the addition of 0.25 and 0.5% *Prosopis farcta* extract enhanced the sensory features even more [98]. As a result, using KG and FG coatings supplemented with *P. farcta* extract should help boost banana commercialization during long-term storage. Tragacanth gum is commonly utilized in the food sector as a polysaccharide food covering. Reduced respiration, dehydration, and enzymatic browning are achieved by using TG as a food coating. Because fruits like bananas deteriorate quickly, TG has also been used as an edible covering material. In comparison to uncoated samples, coated dried banana slices showed reduced shrinkage, higher quality, low water loss, and improved rehydration [60].

The flaxseed mucilage (0.75%, 1% and 1.25%) and xanthan gum (0.5%) was used as a coating material for preservation of Cheddar cheese and it was reported that the coating showed substantial effect on the storage shelf-life of Cheddar cheese [99]. In another work, the effect of varying content of plantego mucilage (15, 20 and 25%) was added to the Arabic gum solution and used as a coating material for chicken breast storage [100]. The meat specimen coated with 25% Plantago showed the lowest value of lipid peroxidation and total bacterial count. The coating significantly improved the life span of chicken breast during storage for 3 weeks.

Taghinia et al. 2021, recently developed intelligent packaging film using *lallemantia iberica* seed mucilage and curcumin for Shrimp freshness indicator [101]. They found that mechanical strength of the film was meaningfully improved while the flexibility reduced in presence of curcumin. The functional film also showed good antioxidant activity as well as antibacterial/mold activity. Moreover, the film showed good pH dependent color indicator properties which used to detect the freshness of Shrimp. It was reported that good correlation between TVBN content of shrimp and color change of the film during storage. The quince seed mucilage-based edible films functionalized with essential oil (oregano or thyme) was used for preservation of rainbow trout fillets [102]. It was reported that the thyme oil incorporated film effectively reduced the microbiological count in the fish fillets during refrigerated storage. Kang et al. 2020, developed okra mucilage and polyvinyl alcohol-based smart color indicator packaging label using anthocyanin [103]. The film showed distinctive color change in pH 2–12 range. The color indicator film was efficiently screen shrimp freshness in real time and the changes in color of the film were clearly identified.

**Figure 4:**
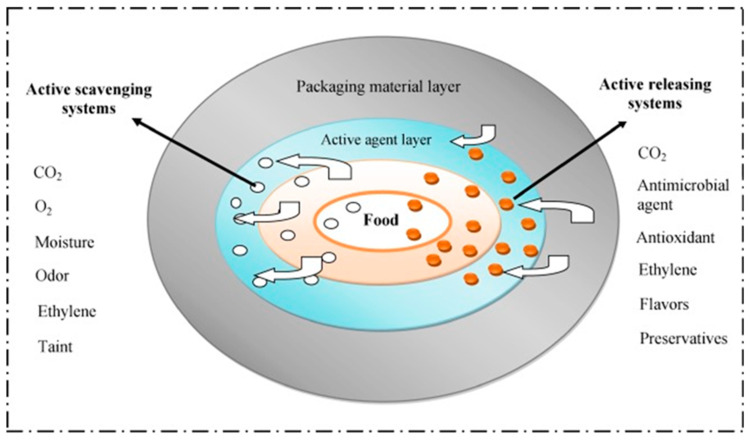
Mechanism of active packaging (Reproduced from Ahmed et al., 2017 [106]).

Edible coatings and films on the surface of food act as a semipermeable membrane barrier which in turn restricts the exchange of gas and moisture [104,105]. The gas and moisture adjust the internal atmosphere of the food, affecting food qualities such as color, sensory quality, firmness, antioxidant activity, etc. Apparently, the different food quality parameters greatly influence food shelf-life [105]. Employing coating and film on food surfaces can effectively reduce the gas and moisture exchange of the food. The inclusion of antioxidant and antimicrobial agents into the food packaging matrix (coating or film) also help improve food shelf life by restricting the growth of unwanted foodborne pathogens, as well as by lowering the oxidation of food [28,104]. The mechanism of an active packaging system is schematically presented in Figure 4. The gas and moisture scavengers are absorbed by the functional active packaging system. The gas scavenger is used to restrict the browning reaction of foods.

The food sector has struggled to preserve and extend the life span of chopped fresh fruits and vegetables. Thus, various food preservation methods have been investigated. Foodborne pathogens have been identified as one of the leading causes of human illness. Therefore, attempts to reduce microbial contamination in fresh fruits and vegetables are inevitable. In this context, applying various coatings on chopped fruits and vegetables could effectively reduce the spread of microbial illnesses. Edible gum-based coatings provide a reductive packaging approach to enhance the life span of foods and prevent postharvest illnesses [107].

## 6. Conclusions and Future Prospective

The manufacturing of bio-based edible coatings and films based on plant-derived natural gums has received enormous attention in recent years. Several researchers have studied and evaluated the potential of plant gums as a possible replacement for synthetic packaging. By altering the proportion of plasticizers and bioactive compounds, edible films with preferred physical characteristics and antioxidant/antimicrobial activities may be developed. Plant-derived, gum-based functional films and coatings are effective in food preservation as they delay ripening, lower respiration, reduce oxidation, hinder microbial development, and carry antioxidants and antimicrobial chemicals that eventually improve the foods’ life span.

Natural gum-based bioactive films and coatings could be beneficial for active packaging purposes, but neat gum-based film exhibited very low mechanical properties and high water affinity compared to synthetic plastic film—which is a primary concern in making active packaging film. Moreover, these gum-based films are not effective for all types of food products owing to their permeable acid, base and water—which can be addressed by mixing with other additives and biopolymers. The mechanical properties of neat natural gum-based films are lower compared to conventional packaging. However, these mechanical (and other physical) properties could be improved by incorporating other biopolymers, bioactive materials, or additives. One of the major tasks facing researchers is to find an appropriate recipe for plant gum biopolymers and additives to achieve optimum parameters for the packaging of food products. Moreover, in most cases, wet methods are produced to develop packaging, which makes commercialization troublesome. Even though natural gum-based materials are promising, further research should emphasize the use of optimum combinations of edible covering materials to improve the nutritional value of foods. Since gum-based edible coatings and films are in the developmental stage, future research should focus on development of prototypes. It is anticipated that researchers will soon be able to address key challenges and advance appropriate skills that will aid industries in upscaling the fabrication of edible films and coatings for food products.

## Figures and Tables

**Table 1:** Types of natural gums used in fabrication solution casting films.

Types of Gums	Additives	Conditions	References
Guar Gum	Chitosan	To make the films, 150 mL of film-forming solutions were put into Teflon plates that were leveled. After that, the plates were dried at 50 °C in ventilated oven with relative humidity of 50%.	[62]
Locus Bean Gum		Before casting into the plate, the temperature was kept at 80 °C for 1 h.	[47]
Tara Gum	Glycerol	TG was suspended in distilled water while being agitated for a period of 3 h at 45 °C. After that, the solution was centrifuged for 3 min at 4000 rpm.	[63]
Basil Seed Gum	Glycerol	At 60 °C, the solution was stirred continuously for 45 min. BSG was cast by pouring the mixture onto a polypropylene plate and drying it for 10 h at 40 °C in a hot-air oven.	[64]
Fenugreek Gum	Glycerol	FG solution (aq.) was prepared. A magnetic stirrer was used to mix the solution at 65 °C for 3 h at 1000 rpm. The nano clay was gently added to the mixture, and it was mixed for another 3 h under the same circumstances.	[55]
Ghatti Gum	Glycerol and Sorbitol	Aqueous solution was prepared at 25 ± 2 °C for 15 min with continuing stirring, then heated at 40 °C.	[58]
Tragacanth and Persian Gum	Glycerol	Centrifugation was used to separate water-insoluble part after being heated to 70 °C and agitated for 10 min.	[65]

**Table 2:** Various plant originated gum based functional films and coatings.

Types of Gums	Added Fillers	Key Result	References
Guar Gum	Curcumin/orange oil	The curcumin addition or cross-linking did not significantly change the thickness of the film. The density of the film was increased while the dissolution of the film in water greatly improved (~50%). The water vapor permeability of the film was reduced and the water contact angle slightly improved (15%). The opacity of the film increased meaningfully without significantly altering the moisture percentage in the film. The antimicrobial action of orange oil and curcumin were retained in films toward *E. coli* and *B. subtills*.	[68]
Locust Bean Gum	Carrageenan/Clay	When the clay concentration was raised, the films’ strength and flexibility values were also improved significantly. The gas permeability ability was also highly influenced by the different content of clay. The thermal degradation of the film was also delayed in presence of clay minerals. The film showed increased antimicrobial activity toward *L. monocytogenes*.	[69]
Locust Bean Gum	Carrageenan	The mixing of κ -carrageenan into LBG increased the films’ barrier characteristics, resulting in a reduction in water vapor permeability (WVP). Moreover, the tensile strength of the k-carrageenan/LBG mix films meaningfully increased (20%) when k-carrageenan/LBG was mixed in 40:60. Actually, the hydrogen bonds interactions among κ-carrageenan and LBG exerted a strong influence on film properties. The moisture content of the film also varied significantly.	[70]
Basil Seed Gum	Oregano essential oil	The thickness of the film was unaltered after adding the essential oil. The WVP was reduced pointedly (~10%) by the inclusion of Oregano essential oil, whereas the moisture content remained unchanged. Contact angle improved pointedly from 48.5 °C to 82 °C, transparency and swelling indexes of edible films were also increased. The film showed strong antioxidant activity in DPPH, ABTS, and FRAP assay. Antimicrobial property of the film was expressively increased against *S. Typhimurium*, *E. coliO157:H7*, *P. aeruginosa*, *S. aureus*, and *B. cereus* in presence of the Oregano essential oil and it was found to be maximum in *B. cereus.*	[71]
Basil Seed Gum	*Zataria* multiflora essential oil	The *Zataria* multiflora essential oil nanoemulsion-incorporated film showed better physical and functional performance. The thickness of the film was slightly increased, while the water solubility was decreased by 20%. The density of the film increased slightly, whereas the mechanical strength of the bioactive film was significantly improved from 20 mPa to 35 mPa in presence of 3 wt% of nanoemulsion. Antimicrobial property of the film was increased in presence of essential oil. The film showed bacteristaric effects on both *E. coli* and *B. cereus*.	[72]
Fenugreek Gum	Nano clays	The incorporation of nanoclays reduced the moisture content of the film and increased tensile strength 4-fold; there was a slight reduction in elongation at break (EB) of the film. The water vapor barrier properties and thermal stability were not greatly altered in presence of nanoclays. Moreover, the composite film showed excellent antimicrobial activity towards food-borne pathogens (*S. aureus*, *L. monocytogenes*, *E. coli O157:H7*, *B. cereus*). The highest zone of inhibition was detected for *L. monocytogenes.*	[55]
Ghatti Gum	Sodium alginate	The mixing of sodium alginate in ghatti gum produced a compatible film. The opacity and WVP of the film were increased after mixing with sodium alginate. The blending of sodium alginate in ghatti gum also improved the mechanical strength of films by 10%. The blend film showed enhanced light barrier properties (65.17%).	[73]
Persian Gum	Sodium caseinate/*Zingiber officinale* extract	The Persian gum/sodium caseinate-based bioactive films tensile strength and EB were significantly improved after mixing *Zingiber officinale* extract. Moreover, the transparency of the film was reduced while the hydrodynamic properties (water resistance, WVP, and water solubility) of the film pointedly improved.	[74]
Tragacanth Gum	Whey protein	The gum tragacanth-added whey protein film was more flexible and less brittle, while the tensile strength of the film was slightly reduced. The thickness and density of the film were decreased, whereas the opacity increased. The total soluble matter of the film was decreased by 20%. The 1.5% gum-included whey protein film showed almost 50% improvement in water vapor barrier properties.	[75]
Carrageenan Gum	Plantago seeds mucilage and red beet extract	The film developed with mucilage (0–20%) and red beet extract (0–10%) showed a decrease in the tensile strength and transparency. The mixing of mucilage enhanced the crystalline property of the film while the extract reduced the crystallinity. The antioxidant performance of the mucilage and extract included film was pointedly improved.	[76]

**Table 3:** Application of gum-based film films and coatings on food preservation.

Types of Gums	Source	Application on Food Product	Observation	References
Guar Gum	*Cyamopsis tetragonolobus*	Roma tomato	Delaying the ripening process at 22 ± 2 °C. The quality of the Roma tomato was better preserved and, most importantly, the bacterial counts found to be lowered.	[92]
Guar Gum	*Cyamopsis tetragonolobus*	Unripe green mangoes	The quality of the green mangoes was better preserved and, most notably, the microbial counts were lowered. As a result, the ripening process of green mangoes was delayed.	[93]
Locust Bean Gum	*Caesalpinia spinosa*	Sausages	The packaging reduced oil content and increased shelf life of the sausage.	[94]
Tara Gum	*Anogeissus latifolia*	Peaches	The gum-based functional packaging lowered growth of yeast and molds on peaches.	[95]
Basil Seed Gum	*Ocinum basilicum*	Strawberries	The basil seed gum-based film helped to extend shelf life, reduce mass losses, and maintain the nutritional value of the strawberries.	[96]
Fenugreek Gum	*Trigonella foenum-groecum*	Guava	The packaging reduced weight loss and enhanced shelf life of guava.	[97]
Ghatti Gum	*Anogeissus latifolia*	Grapes	The antioxidant activity of the film helped to improve the preharvest quality of the grapes.	[57]
Persian Gum	Wild almond tree	Banana	The persian gum-based film effectively reduced microbial growth on bananas during storage.	[98]
Tragacanth Gum	Astragalus	Apple slices	The packaging decreased respiration, dehydration, and enzymatic browning of apple slices, which was effective to improve the shelf life.	[60]
Xanthan Gum and Flaxseed Mucilage	Sugar and *Xanthomonas campestris* bacteria	Cheddar cheese	Coating the cheddar cheese with xanthan gum and flaxseed mucilage exhibited noteworthy effects on chemical properties such as acidity, pH, fats, dry matters, and moisture of cheese during storage for 3 months.	[99]
Arabic Gum and plantago Seeds Mucilage	Acacia senegal and Acacia seyal	Chicken breast	The coating improved the shelf life of chicken breast by delaying the spoilage during storage at 4 °C.	[100]

## Data Availability

Not applicable.
